# High Prevalence of HIV, HCV, HBV and Co-Infection and Associated Risk Factors among Injecting Drug Users in Yunnan Province, China

**DOI:** 10.1371/journal.pone.0042937

**Published:** 2012-08-16

**Authors:** Yan-Heng Zhou, Zhi-Hong Yao, Feng-Liang Liu, Hong Li, Li Jiang, Jia-Wu Zhu, Yong-Tang Zheng

**Affiliations:** 1 Key Laboratory of Animal Models and Human Disease Mechanisms of Chinese Academy of Sciences & Yunnan Province, Kunming Institute of Zoology, Chinese Academy of Sciences, Kunming, China; 2 Yunnan Center for Disease Control and Prevention, Kunming, China; 3 The Graduate School of the Chinese Academy of Sciences, Beijing, China; Duke University, United States of America

## Abstract

**Objective:**

To estimate the prevalence of HIV, HCV, HBV and co-infection with 2 or 3 viruses and evaluate risk factors among injecting drug users (IDUs) in Yunnan province, China.

**Methods:**

2080 IDUs were recruited from 5 regions of Yunnan Province, China to detect the infection status of HIV, hepatitis B virus (HBV) and hepatitis C virus (HCV). Statistical analysis was performed to evaluate risk factors related to HIV, HCV and HBV infections.

**Results:**

The infection rates among all participants were 25.5% for HIV, 77.7% for HCV, 19.2% for HBV, 15% for HIV/HCV, 0.3% for HIV/HBV, 7.8% for HCV/HBV and 7.1% for HIV/HCV/HBV. The prevalence of virus infection varied widely by region in Yunnan of China. Statistical analyses indicated that high prevalence of HIV and HCV among IDUs was positively associated with the duration of drug injection and sharing needles/syringes; besides, HCV infection was associated with the frequency of drug injection.

**Conclusions:**

HIV, HCV, HBV infections and co-infections were still very prevalent among IDUs in Yunnan province because of drug use behaviors.

## Introduction

Yunnan is one of the provinces hardest hit by HIV epidemic in China, since the first large HIV outbreak occurred in 1989 among injecting drug users (IDUs) in Dehong prefecture [1]–[4]. In Yunnan, the cumulative number of HIV positives reported at the end of October 2011 was 93, 567, including 25, 698 AIDS cases and 14,340 recorded deaths. According to sentinel surveillance data, HIV prevalence mainly concentrated on two high-risk populations: IDUs and female sexual workers (FSWs). 53.2% HIV positive cases were IDUs, and 18.6% were FSWs [5]. Although HIV infection through heterosexual contact has increased steadily from less than 5% in 1996 to up to 20% recently, intravenous drug use is still the dominate transmission mode of HIV [4]. A long history of opium/heroin trade and high prevalence of illicit drugs, majority of which are trafficked from “golden triangle”, have partly accounted for the high prevalence of HIV infection in Yunnan, especially among IDUs [6]. The data showed that HIV in Yunnan has primarily spread through intravenous drug use with a high annual incidence rate of 2.2%–8.0% [7], and the provincial average prevalence among IDUs fluctuated between 21.2% and 27.8% through 2004 [3]. Moreover, the rate of HIV infection among IDUs was reported as high as 74.5% [8]. However, HIV prevalence varies from region to region in Yunnan Province. Sentinel surveillance data in 2004 indicated that HIV prevalence among IDUs was 60.4% in Kaiyuan city, 41% in Yingjiang county, 21.5% in Qujing prefecture, 4.6% in Zhaotong prefecture and 2.9% in Baoshan prefecture [9]. Furthermore, these ratios reported recently still reveal uneven prevalence in different regions. By the end of 2007, in several regions including Yingjiang county and Kaiyuan city, HIV prevalence among IDUs was also higher than 40% [8]. Another study demonstrated HIV prevalence among Kaiyuan's IDUs was as high as 59.9% in 2007 [10]. From 1992 to 2009, 3591 IDUs were tested for HIV and the average infection rate was 3.15% in Baoshan [11].

Besides HIV, the prevalence of hepatitis B virus (HBV) and hepatitis C virus (HCV) infection among IDUs is also alarming. Previous studies conducted in 2007 showed 68.1% IDUs were infected with HCV in Yunnan [12]. Another study estimated in 2009 the rate of HCV positive was 83.33% among Yunnan IDUs [13]. Tian et al found the infection rate among Yunnan IDUs was 69.7% for HCV and 36.45% for HBV [14]. Meanwhile, HCV and HBV infection among IDUs in Dehong was 35.3% and 52.9%, respectively [15]. Due to sharing the similar transmission mode, co-infection with HIV and hepatitis viruses is quite frequent amongst IDUs sharing needles/syringes with each other [16]. It was indicated the rate of sharing injecting equipment was as high as 59.4% among HIV-infected IDUs in Yunnan [9], and 93.3% to 99.3% of them were co-infected with HCV [12], [17]. High prevalence of co-infection with HCV (92%) or HBV (71%) among HIV positive IDUs was also reported in Ruili county, Yunnan province [18].

Previous studies have confirmed that HIV co-infection can accelerate the clinical course of chronic HCV or HBV infection and increase the risks of liver cirrhosis, hepatocellular carcinoma (HCC) and decompensated liver disease [19], [20]. Although the effects of HCV or HBV on HIV are controversial, some studies have shown HIV positive patients co-infected with HCV and/or HBV have more rapid progression of AIDS and related death compared with patients without co-infections [21]. Additionally, co-infection with HCV and HBV is not uncommon among IDUs, and patients with dual HCV and HBV infection have more severe liver disease with increasing risk for progression to HCC [22]. Therefore, it is indispensable to investigate and ascertain the prevalence of the co-infection of HIV and HCV and/or HBV, or HCV and HBV to understand the true burden of disease among IDUs.

HIV infection among IDUs has been well recognized in Yunnan, China. However, limited data are available on the prevalence of HCV, HBV and co-infection with HIV and HCV and/or HBV, or HCV and HBV, especially in respective prefecture. Therefore, a cross-sectional study was conducted to estimate the prevalence of HIV, HCV, HBV and co-infection with 2 or 3 viruses and evaluate risk factors among IDUs in Yunnan province.

## Methods

### Study population

A cross-sectional study was conducted among IDUs recruited from communities, free HIV Voluntary Counseling and Testing (VCT), needle and syringe programs (NSP), or Methadone maintenance treatment programs (MMT) with the assistance of local Centers for Disease Control and Prevention (CDC) in 5 regions of Yunnan province between March 2009 and October 2011. Those who met the following criteria were selected as study subjects: 1) being age ≥16 years, 2) had a history of injecting drug use, 3) be able to provide informed consent. After written consent, all eligible participants were interviewed privately and confidentially with a pre-code, structured questionnaire, which was developed after reviewing published literatures on risk factors for HIV, HCV and HBV infection. It assessed demographic characteristics (e.g., age, ethnicity and marital status), risk behaviors including the duration of drug injection, needles/syringes sharing, the number of sexual partner and ever having a history of sexually transmitted diseases (STD). All variables can be found in Table 1. Volunteers could end the interview at any time or refuse to answer any question(s), and decline to donate blood without any negative consequence. After completion of the interview, subjects were asked to donate venous blood. However, blood samples were abandoned to be collected from IDUs who refuse to answer any question(s) in questionnaire. At last, a total of 2080 samples were collected and stored at −70°C until use according to standard procedures.

**Table 1:** Demographic and risk behavior characteristics of the IDUs in five study sites.

Variable	Total No.(%) N = 2080	Zhaotong No.(%) N_1_ = 570	Qujing No.(%) N_2_ = 229	Kaiyuan No.(%) N_3_ = 329	Baoshan No.(%) N_4_ = 472	Yingjiang No.(%) N_5_ = 480	chi-square test *p* ^ 1^
Age							<0.001^a^
N(respondents)	2071	569	225	329	472	476	
mean(95%CI)	31.7 (31.4,32.0)	28.9 (28.4,29.4)	29.2 (28.3,30.1)	37.1 (36.4,37.8)	32.0 (31.4,32.7)	32.1(31.3,32.9)	
Gender							<0.001
Male	1937 (93.3)	558 (97.9)	228 (100)	242 (73.6)	433 (92.1)	476 (99.2)	
Female	140 (6.7)	12 (2.1)	0 (0)	87 (26.4)	37 (7.9)	4 (0.8)	
Ethnicity							<0.001
Han	1557 (75.0)	435 (76.3)	217 (94.8)	263 (79.9)	440 (93.4)	202 (42.3)	
Hui	164 (7.9)	115 (20.2)	6 (2.6)	32 (9.7)	11 (2.3)	0 (0)	
Dai	206 (9.9)	0 (0)	0 (0)	0 (0)	7 (1.5)	199 (41.7)	
Jingpo	64 (3.1)	0 (0)	0 (0)	0 (0)	3 (0.6)	61 (12.8)	
Yi	50 (2.4)	17 (3.0)	4 (1.7)	24 (7.3)	4 (0.8)	1 (0.2)	
other	35 (1.7)	3 (0.5)	2 (0.9)	10 (3.0)	6 (1.3)	14 (2.9)	
Occupation							<0.001
Farmer	743 (38.0)	95 (17.8)	70 (35.5)	4 (1.2)	180 (42.3)	394 (84.0)	
Employed	323 (16.5)	105 (19.6)	46 (23.4)	41 (12.5)	106 (24.9)	25 (5.3)	
Unemployed	889 (45.5)	335 (62.6)	81 (41.1)	283 (86.3)	140 (32.9)	50 (10.7)	
Marriage status [n (%)]							<0.001
Single	909 (43.8)	275 (48.2)	107 (46.9)	140 (42.6)	193 (41)	194 (40.5)	
Married/live with partner	916 (44.1)	242 (42.5)	105 (46.1)	134 (40.7)	232 (49.3)	203 (42.4)	
Divorced	252 (12.1)	53 (9.3)	16 (7)	55 (16.7)	46 (9.8)	82 (17.1)	
Education level							
None	141 (6.8)	48 (8.4)	12 (5.4)	7 (2.1)			
Primary	682 (33.0)	203 (35.7)	81 (36.3)	66 (20.1)			
Secondary	942 (45.6)	241 (42.4)	104 (46.6)	161 (49.1)			
High school/university	303 (14.7)	77 (13.5)	26 (11.7)	94 (28.7)			
Age of first drug injection							
N(respondents)	1928	555	188	288			
mean(95%CI)	24.7 (24.4,25.0)	23.5 (23.0,24.0)	23.1 (22.3,23.9)	25.2 (24.4,26.0)			
Years of drug injection							
N(respondents)	1936	556	192	288			
mean(95%CI)	6.8 (6.6,7.0)	5.4 (5.0,5.8)	5.9 (5.2,6.6)	11.6 (11.0,12.2)			
Main routes of drugs use							
Injecting only	1530 (89.1)	509 (89.6)	189 (85.9)	NA			
Non-injecting	187 (10.9)	59 (10.4)	31 (14.1)	NA			
Frequency of drug injection							
mean (95%CI)	3.0 (2.9,3.1)	2.7 (2.6,2.8)	3.1 (2.9,3.3)	NA			
≤1 time/d	164 (10.0)	63 (11.3)	12 (6.2)	NA			
1–3 times/d	563(34.4)	227(40.7)	73(38)	NA			
≥3times/d	910(55.6)	268(48.0)	107(55.7)	NA			
Frequency of needles used							<0.001
mean(95%CI)	1.9 (1.8,2.0)	1.6 (1.5,1.7)	1.5 (1.4,1.6)	NA	1.6 (1.44,1.68)	2.6 (2.3, 2.9)	
Once	863 (52.5)	244 (43.6)	115 (60.2)	NA	245 (57.9)	187 (39.8)	
>1 time	781 (47.5)	316 (56.4)	76 (39.8)	NA	178 (42.1)	283 (60.2)	
Needles/syringes sharing							<0.001
Yes	556 (33.7)	232 (41.3)	87 (46.0)	NA	62 (14.5)	175 (37.1)	
No	1094 (66.3)	330 (58.7)	102 (54.0)	NA	365 (85.5)	297 (62.9)	
NO of sexual partner							<0.01
mean(95%CI)	5.2 (4.6,5.8)	5.6 (4.6,6.6)	4.6 (3.5,4.7)	NA	5.7 (4.5,6.9)	4.6 (3.1,6.1)	
0	204(12.6)	47(9.2)	39(18.8)	NA	37(8.3)	81(17.8)	
1	515(31.8)	128(25)	47(22.6)	NA	194(43.4)	146(32.2)	
>1	902(55.6)	337(65.8)	122(58.7)	NA	216(48.3)	227(50)	
History of sexual transmission disease							<0.01
No	1430(84.2)	428(76.8)	153(70.5)	NA	436(93.8)	413(89.8)	
Yes	269(15.8)	129(23.2)	64(29.5)	NA	29(6.2)	47(10.2)	

Note: Numbers may not add up to total because not all participants answered all questions. *p* ^1^ value showed the difference among 5 study sites.

NA: not available a: Kruskal-Wallis test.

### Ethical clearance

Ethical approval for the study and the informed consent process were approved by the Ethics Committee of Kunming Institute of Zoology, Chinese Academy of Sciences. The research was conducted in accordance with basic principles of the Helsinki declaration and the relevant international rules.

### Viral infection assays

Blood samples were used for testing HIV, HCV and HBV status. HIV infection was identified using the fourth generation of enzyme-linked immunosorbent assay testing kit (ELISA, Wantai Biological Pharmacy Enterprise Co., Ltd, Beijing, China) and confirmed by another ELISA kit (Shanghai Kehua Bio-engineering Co., Ltd., Shanghai, China). Specimens with positive results in both ELISA assays were considered positive for HIV. ELISA kits were used to test the presence of anti-HCV and HBsAg (Shanghai Kehua Bio-engineering Co., Ltd., China) respectively.

HIV/HCV, HIV/HBV, HCV/HBV co-infection and HIV/HCV/HBV triple infections were defined as previously [23], namely positive HIV and HCV serology, positive HIV and HBV serology, positive HCV and HBV serology, positive HIV, HCV and HBV serology, respectively.

### Data analysis

Data was inputted in excel and transferred into the R program for data exploration and analysis. The demographic data of participants were described in percentage and mean. Prevalence of infections was calculated by regions. Significance of difference was initially assessed with the chi-square (χ2) test and Kruskal-Wallis test. As potential confounding was considered among behaviors, multivariate logistic regression analyses was used to identify independent risk factors for infections. 95% confidence interval (CI) and odd ratios (ORs) were calculated. P value <0.05 was used to indicate statistical significance.

## Results

### Description of the study population

A total of 2080 IDUs were recruited, with 570 were from Zhaotong, 229 from Qujing, 329 from Kaiyuan city, Honghe prefecture, 472 from Baoshan, 480 from Yingjiang county, Dehong prefecture. The mean age of study cohorts was 31.7 years old. Male (93.3%) and Han ethnicity (75.0%) constituted the majority, and 45.5% IDUs were unemployed and 43.8% were single. These demographic characteristics varied dependent on regions and the differences were significant (p<0.001, Table 1). Drug use and sexual behaviors were also shown in the Table 1. Totally, most IDUs (89.1%) were injecting only. The mean duration of drug injection was 6.8 years ranging from 5.4 in Zhaotong to 11.6 in Kaiyuan. More than half of them (55.6%) injected drugs ≥3 time a day and 33.7% reported sharing needles/syringes with others. 55.6% had more than 1 sexual partner. Furthermore, 15.8% IDUs had the history of STD, however, none of them reported having sex with a person of the same sex (data not shown in Table 1). As demographic characteristics, there were significant differences in risk behaviors among IDUs from 5 study sites (p<0.001 or p<0.01).

### Prevalence of HIV, HCV and HBV infection

**Figure 1:**
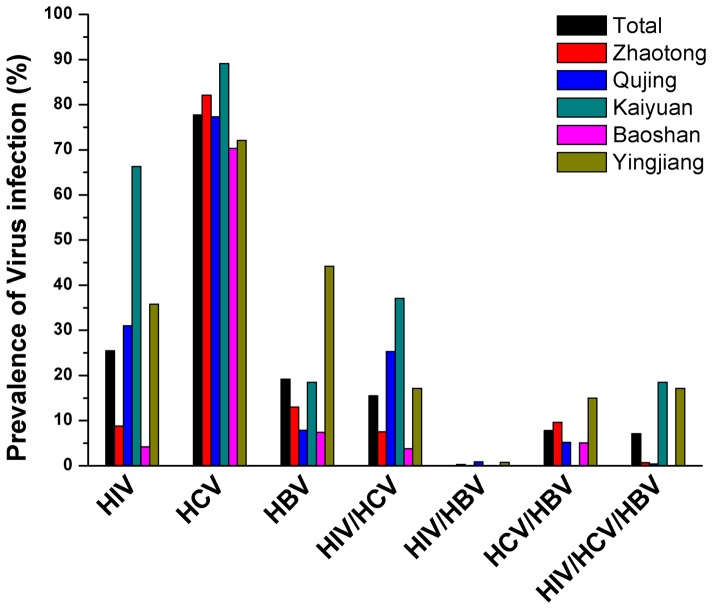
Prevalence of HIV, HCV, HBV infection and co-infection among 5 study sites.

As our previous study [23], HCV (77.7%) is more prevalent than HIV (25.5%) and HBV (19.2%) among all IDUs (Table 2, Figure 1). HIV infection in Kaiyuan (66.3%) was the most prevalent. The difference of HIV prevalence among 5 regions was statistical significant (p<0.001). Like HIV, there was also geographical distinction in HCV and HBV prevalence (p<0.001). It was notable that Baoshan not only had the lowest HIV infection but also HCV and HBV infection (70.3% and 7.4%, respectively). However, Kaiyuan had the highest ratio of HCV (89.1%), while Yingjiang was the most prevalent region of HBV (44.2%) among 5 study sites (Table 2).

**Table 2:** Prevalence of HIV, HCV, HBV infection and co-infection of two or three viruses among studied cohorts.

	Total (N = 2080) n(%)	Zhaotong (N_1_ = 570) n(%)	Qujing (N_2_ = 229) n(%)	Kaiyuan (N_3_ = 329) n(%)	Baoshan (N_4_ = 472) n(%)	Yingjiang (N_5_ = 480) n(%)	chi-square test *P* ^2^ value
**HIV**	**531 (25.5)**	**50 (8.8)**	**71 (31.0)**	**218 (66.3)**	**20 (4.2)**	**172 (35.8)**	**<0.001**
**HCV**	**1616 (77.7)**	**468 (82.1)**	**177 (77.3)**	**293 (89.1)**	**332 (70.3)**	**346 (72.1)**	**<0.001**
**HBV**	**400 (19.2)**	**74 (13.0)**	**18 (7.9)**	**61 (18.5)**	**35 (7.4)**	**212 (44.2)**	**<0.001**
**HIV/HCV**	**323 (15.5)**	**43 (7.5)**	**58 (25.3)**	**122 (37.1)**	**18 (3.8)**	**82 (17.1)**	**<0.001**
**HIV/HBV**	**6 (0.3)**	**0 (0)**	**2 (0.9)**	**0 (0)**	**0 (0)**	**4 (0.8)**	**0.02**
**HCV/HBV**	**163 (7.8)**	**55 (9.6)**	**12 (5.2)**	**0 (0)**	**24 (5.1)**	**72 (15.0)**	**<0.001**
**HIV/HCV/HBV**	**148 (7.1)**	**4 (0.7)**	**1 (0.4)**	**61 (18.5)**	**0 (0)**	**82 (17.1)**	**<0.001**

*p* ^2^ value showed the difference among 5 study sites.

### Prevalence of HIV/HCV, HIV/HBV, HCV/HBV and HIV/HCV/HBV co-infection

The prevalence of HIV/HCV, HIV/HBV, HCV/HBV and HIV/HCV/HBV among IDUs was 15.5%, 0.3%, 7.8% and 7.1%, respectively (Table 2, Figure 1). The ratio of co-infection with 2 or 3 viruses varied by regions (p<0.001 or p = 0.02). In brief, the highest prevalence of HIV/HCV co-infection (37.1%) and triple infections (18.5%) was both seen in Kaiyuan where no one co-infected with HIV and HBV. Yingjiang showed the most prevalent of HCV/HBV (15%). Qujing (0.9%) and Yingjiang (0.8%) were only two regions existing HIV/HBV co-infection.

### Risk behaviors associated with HIV, HCV and HBV infection

In order to analyze the relationship between drug use and sexual behaviors and viruses infection, we firstly did univariate analysis. The results indicated that risk factors, such as long time of drug injection (≥5 years), frequent injecting drug and needles/syringes sharing as well as STD, were significantly associated with HIV and HCV infection (p<0.001 or p = 0.003), but we didn’t find any drug use and sexual behaviors in our study might be risk factors of HBV infection (Table 3). Besides, it was shown high ratio of injecting during drug usage was easily infected with HCV (p<0.001, Table 3). Secondly, we performed multivariate analyses to further make sure risk factors. As a result, we obtained almost consistent results, except that frequent injecting drug and STD were no more the risk factors associated with HIV, and STD was not associated with HCV infection (Table 4). In brief, both univariate analysis and multivariate analysis indicated that long time of drug injection (≥5 years) and needles/syringes sharing were associated with HIV and HCV infection, and frequent injecting drug (>1 time/d) was also associated with HCV infection besides.

**Table 3:** Risk behaviors and the prevalence of HIV, HCV and HBV infection in studied cohorts.

Variables	Total	HIV		HCV		HBV	
		n(%)	*P* * value	n(%)	*P* * value	n(%)	*P* * value
Main route of drugs use			0.104		<0.001		0.051
Non-injecting	187	25 (13.4)		108 (57.8)		26 (13.9)	
Injecting only	1530	283 (18.5)		1206 (78.8)		309 (20.2)	
Years of injecting drug use			<0.001		<0.001		0.417
<5 yr	796	81 (10.2)		540 (67.8)		165 (20.7)	
≥5 yr	852	216 (25.4)		752 (88.3)		163 (19.1)	
Frequency of drug injection			<0.001		<0.001		0.089
≤1 time/d	164	20 (12.2)		93 (56.7)		43 (26.2)	
1–3 times/d	564	81(14.4)		423(75.1)		105(18.6)	
≥3times/d	909	198(21.8)		776(85.3)		176 (19.4)	
Needles/syringes sharing			<0.001		<0.001		0.319
No	1094	112 (10.2)		790 (72.2)		210 (19.2)	
Yes	556	185 (33.3)		506 (91)		119 (21.4)	
No of sexual partner			0.137		0.523		0.268
1	515	94 (18.3)		379 (73.6)		111 (21.6)	
>1	902	136 (15.1)		679 (75.3)		171 (18.9)	
History of sexual transmission disease			0.003		<0.001		0.63
No	1699	238 (14.0)		1076 (63.3)		280 (16.5)	
Yes	269	57 (21.2)		216 (80.3)		48 (17.8)	

* *P* value for chi-square test

**Table 4:** Risk behaviors associated with HIV, HCV and HBV infection in study population in logistic regression*.

Variables	HIV			HCV			HBV		
	COR (95% CI)	AOR (95% CI)	*P* value	COR (95% CI)	AOR (95% CI)	*P* value	COR (95% CI)	AOR (95% CI)	*P*
Main route of drugs use			0.212			0.839			0.991
Non-injecting	1	1		1	1		1	1	
Injecting only	1.78 (0.96,3.29)	1.5 (0.78,2.88)		1.25 (0.8,1.94)	1.05 (0.64,1.73)		1.04 (0.64,1.69)	1.00 (0.59,1.71)	
Years of injecting drug use			<0.001			<0.001			0.07
>5 yr	1	1		1	1		1	1	
≥5 yr	3.11 (2.29,4.23)	2.06 (1.45,2.94)		3.37 (2.57,4.42)	2.26 (1.66,3.08)		0.98 (0.76,1.27)	0.75(0.55,1.02)	
Frequency of drug injection			0.208			<0.001			0.711
≤1 time/d	1	1		1	1		1	1	
1–3 times/d	1.17 (0.65,2.09)	0.83 (0.44,1.57)	0.566	2.18 (1.48,3.21)	1.69 (1.12,2.55)	0.012	0.71 (0.46,1.11)	0.84 (0.52,1.36)	0.476
≥3times/d	2.09 (1.21,3.62)	1.14 (0.62,2.1)	0.682	4.4 (3,6.44)	2.82 (1.86,4.29)	<0.001	0.75 (0.49,1.13)	0.82 (0.51,1.31)	0.409
Needles/syringes sharing			<0.001			<0.001			0.878
No	1	1		1	1		1	1	
Yes	4.48 (3.35,5.99)	3.93 (2.86,5.4)		3.6 (2.59,5.01)	2.63 (1.85,3.75)		1.1 (0.84,1.44)	1.02 (0.75,1.39)	
No. of sexual partner			0.247			0.45			0.824
1	1	1		1	1		1	1	
>1	0.93 (0.69,1.26)	0.8 (0.55,1.16)		1.07 (0.82,1.41)	0.88 (0.64,1.22)		0.86 (0.65,1.13)	1.04 (0.74,1.45)	
Have history of STD			0.359			0.99			0.712
No	1	1					1	1	
Yes	1.38 (0.97,1.95)	1.21 (0.81,1.81)		1.46(1.01, 2.11)	0.99(0.66, 1.51)		0.88 (0.61,1.26)	1.08 (0.72,1.6)	

**Note:** * 1410 IDUs entered in the model of logistic regression; Adjusted by all independent variables.

COR, crude odds ratio; AOR, adjusted odds ratio; *P* value for Likelihood Ratio test.

## Discussion

HIV infection among IDUs has been well recognized in Yunnan province, China [1]–[8]. However, there are limited data on the prevalence of HCV, HBV and co-infection with HIV and HCV and/or HBV, or HCV and HBV among IDUs, especially among them from respective prefecture in Yunnan. It is well known that designing an effective management to control the prevalence of HIV, HCV and HBV among IDUs is a great challenge for health authorities. Therefore, grasping the true burden of disease and associated risk factors among IDUs seem to be the top priority recently. Our study represented the first large investigation to study the HIV, HCV, HBV and co-infection epidemic and assess related risk factors among IDUs in Yunnan province. The results showed high prevalence of HIV (25.5%), HCV (77.7%), HBV (19.2%) and uncommon co-infection of HIV/HCV (15.5%), HIV/HBV (0.3%), HCV/HBV (7.8%), HIV/HCV/HBV (7.1%) among IDUs.

It was obvious that HIV prevalence among IDUs was remarkably high, almost two times higher than national infectious level (12.55%) [24]. IDUs are always one of the most effected groups in Yunnan since the first large HIV outbreak occurred among them in 1989. Initially, HIV infection rate among IDUs had been up to 100%, while the digit reduced to 40% in 2006, 28.7% in 2007, and then remained near that level [7], [8]. However, the severity of HIV prevalence in different regions was still different. By the end of 2004, the survey data from sentinel sites throughout the province indicated that HIV prevalence among IDUs from 5 prefectures including Dehong, Chuxiong, Lincang, Honghe and Wenshan had surpassed 40%. Compared with them, other prefectures such as Qujing (21.5%) Zhaotong (4.6%) and Baoshan (2.9%) had relative lower prevalence [9]. Furthermore, a prior study conducted in 2007 in Kaiyuan city also demonstrated HIV prevalence among IDUs was as high as 59.9% [22]. Another study also revealed the high prevalence of HIV among IDUs in Dehong. It was estimated that there were at least 15000 IDUs and 45.4% were infected with HIV in 2004, 38.4% in 2008 [25], [26]. Our data showed this different severity consistently. Dehong prefecture and Honghe prefecture are in the proximity to the border with Myanmar and Vietnam, respectively. It's well known cross-border travel and commerce including illicit drug trade are very common, which may be responsible for the high prevalence among IDUs in these prefectures.

Interestingly, although Baoshan locates near to Yingjiang, Dehong prefecture, it has relative lower HIV infection rate among IDUs (4.2%) compared with that in the latter (35.8%). One reason for the substantial regional heterogeneity in HIV prevalence may be the difference in drug use behaviors between two regions. Our study found HIV infection was strongly associated with needles/syringes sharing, and the ratio of neddles/syringes sharing among Yingjiang's IDUs (37.1%) was significant higher than among baoshan's IDU, which may be the reason of low HIV epidemic in Baoshan. It seems to be the first time to interpret uneven prevalence of HIV among IDUs between Baoshan and Yingjiang. In addition, we estimated that Zhaotong prefecture has a great potential for HIV spread among its IDUs since high prevalence of risk factors like needle sharing (41.3%) [27].

On the other hand, it is worthy to note that there is a high prevalence rate of HCV among overall IDUs (77.7%), and even in IDUs from Baoshan (70.3%) and Zhaotong (82.1%), where HIV infection among them is relative low (Table 2, Figure 1). Previous studies have demonstrated that the prevalence rate of HCV is up to 10 times higher than that of HIV, and HCV prevalence could be an indicator of HIV risk among IDUs [28], [29]. Our results showed that there is a high prevalence of HCV infection but low HIV prevalence both in Zhaotong and Baoshan. Therefore, we estimated that either HIV prevalence might have been effectively controlled or a looming HIV epidemic may be appearing among IDUs in these two regions. As is well known, China is a high prevalence area of HBV with a general seroprevalence of 5.84% [30]. Much high HBV prevalence was identified among IDUs (19.2%) in this study. This may imply more effective strategies are needed to control HCV and HBV infections among IDUs.

Long duration of injecting and needle sharing are believed to be the major risk factors for HIV and HCV infections among IDUs [25], [31]–[33]. Our research confirmed their results. Furthermore, among our study population, 57.1% IDUs had injected drugs for ≥5 years, 33.7% had shared needles and 55.6% had reported injecting drugs ≥3 times a day. In addition, we also found IDUs who were old, minority ethnicity, farmers or had primary education were more likely to infect HIV. The factors significantly associated with HCV were older age, unemployed. For HBV, the factors were older age, minority ethnicity and farmer, while higher education was found to be a protective factor (data not shown). However, our study failed to establish association between HBV and drug use or sexual behaviors. This could be partly explained by the immunity to HBV before injecting or heterosexual contact was initiated. It was reported that HBV vaccine had been initiated from 1985 in China, and the immunization coverage of it among children aged from 1 to 3 years was 70.34% in Yunnan [34], while significant difference of the coverage was also found in different regions with higher rate in urban and lower rate in rural areas, which may be the evidence for the higher prevalence of HBV among farmers in our study. These findings suggest that prevention and intervention should be paid more attention to IDUs who were older, minority ethnicity, farmer/unemployed to control high risk behaviors. On the other hand, the first pilot of NSP was launched in Yunnan province in 1999 [24]. It is estimated NSP has averted approximately 16–20% of the potential HIV cases since 2002 [6]. Despite of the large investment and positive achievement in NSP, less than 25% IDUs obtain their injecting equipment through NSP and 45% remains sharing injecting equipment [6]. Hence, there is much more room for NSP to control high risk drug use behaviors among IDUs.

There is a primary limit in our study. Drug use and sexual behaviors of our subjects are self-reported. This may result in missing values and untruthful responses. Because drug use is illegal in our country and discriminated in the society, drug users are likely to hide their activities even though we ensured that there was no threat of prosecution. This may be the reason why we did not get the information of frequency of injecting drugs and whether sharing needle or sexual behavior information among Kaiyuan IDUs. Therefore, we used the data of risk behaviors in other four sites except that in Kaiyuan to assess the association between them with HIV, HCV and HBV infection. Nevertheless, our results are consistent with previous studies. We believe our study have great implication in future efforts on the transmission of HIV, HCV and HBV among IDUs and from them to general population in Yunnan province.

In conclusion, high prevalence of HIV as well as HCV, HBV infections and co-infections was still found among IDUs in Yunnan accompanying with high risk behaviors. These findings underscore the urgent need to improve harm-reduction interventions such as NSP and more effective treatments including launching more education among IDUs who were old, farmers/unemployed and minority ethnicity to control HIV, HCV and HBV infection and transmission among IDUs, especially among them from Zhaotong and Baoshan, where HIV prevalence has the potential growth. Much more attention should also be paid to the high prevalence of co-infections including HIV/HCV, HCV/HBV, HIV/HCV/HBV among IDUs.
